# G protein-gated inwardly rectifying potassium channel subunits 1 and 2 are down-regulated in rat dorsal root ganglion neurons and spinal cord after peripheral axotomy

**DOI:** 10.1186/s12990-015-0044-z

**Published:** 2015-07-22

**Authors:** Chuang Lyu, Jan Mulder, Swapnali Barde, Kristoffer Sahlholm, Hugo Zeberg, Johanna Nilsson, Peter Århem, Tomas Hökfelt, Kaj Fried, Tie-Jun Sten Shi

**Affiliations:** School of Life Science and Technology, Harbin Institute of Technology, 150001 Harbin, China; Department of Neuroscience, Karolinska Institutet, 171 77 Stockholm, Sweden; Department of Neuroscience, Science for Life Laboratory, Karolinska Institutet, 171 77 Stockholm, Sweden

**Keywords:** Axonal transport, Ca^2+^-binding proteins, CGRP, Galanin, GIRK channel, Nerve injury, Neuropathic pain, Neuropeptides, NPY, Nociceptor, Somatostatin

## Abstract

**Background:**

Increased nociceptive neuronal excitability underlies chronic pain conditions. Various ion channels, including sodium, calcium and potassium channels have pivotal roles in the control of neuronal excitability. The members of the family of G protein-gated inwardly rectifying potassium (GIRK) channels, GIRK1–4, have been implicated in modulating excitability. Here, we investigated the expression and distribution of GIRK1 and GIRK2 in normal and injured dorsal root ganglia (DRGs) and spinal cord of rats.

**Results:**

We found that ~70% of the DRG neurons expressed GIRK1, while only <10% expressed GIRK2. The neurochemical profiles of GIRK1- and GIRK2-immunoreactive neurons were characterized using the neuronal markers calcitonin gene-related peptide, isolectin-B4 and neurofilament-200, and the calcium-binding proteins calbindin D28k, calretinin, parvalbumin and secretagogin. Both GIRK subunits were expressed in DRG neurons with nociceptive characteristics. However, while GIRK1 was widely expressed in several sensory neuronal subtypes, GIRK2 was detected mainly in a group of small C-fiber neurons. In the spinal dorsal horn, GIRK1- and -2-positive cell bodies and processes were mainly observed in lamina II, but also in superficial and deeper layers. Abundant GIRK1-, but not GIRK2-like immunoreactivity, was found in the ventral horn (laminae VI–X). Fourteen days after axotomy, GIRK1 and GIRK2 were down-regulated in DRG neurons at the mRNA and protein levels. Both after axotomy and rhizotomy there was a reduction of GIRK1- and -2-positive processes in the dorsal horn, suggesting a presynaptic localization of these potassium channels. Furthermore, nerve ligation caused accumulation of both subunits on both sides of the lesion, providing evidence for anterograde and retrograde fast axonal transport.

**Conclusions:**

Our data support the hypothesis that reduced GIRK function is associated with increased neuronal excitability and causes sensory disturbances in post-injury conditions, including neuropathic pain.

**Electronic supplementary material:**

The online version of this article (doi:10.1186/s12990-015-0044-z) contains supplementary material, which is available to authorized users.

## Background

G protein-gated inwardly rectifying K^+^ (Kir3/GIRK) channels belong to the super-family of proteins known as inward rectifier K^+^ (Kir) channels (Kir1–Kir7) and are mainly found in the nervous system and heart [1, 2]. Kir channels pass current most efficiently at membrane voltages negative to potassium’s reversal potential, thus allowing large inward flux of potassium ions. Although not fully understood, the mechanism behind the rectification involves a high-affinity block by endogenous polyamines and magnesium ions at depolarized potentials, resulting in a reduction of outward current [1, 2].

Functional GIRK channels in mammals are composed of homo- or heterotetramers consisting of subunits GIRK1–4 (including splice variants of GIRK1 and GIRK2) [3–7]. However, it is uncertain whether all GIRKs are functional in native tissue. GIRK1 does not form functional homomultimeric channels, probably due to absence of an endoplasmic export signal [8–10]. The activation of inhibitory G-protein-coupled receptors (GPCRs) [2] opens GIRK channels via Gβγ subunits released from G proteins. This can suppress neuronal excitability and inhibit transmitter release [11]. Numerous GPCRs, including opioid, adrenergic, dopaminergic, GABA_B_, muscarinic cholinergic, cannabinoid, endothelin (B), somatostatin and galanin receptors, are functionally coupled with GIRK channels in the nervous system [12–17]. GIRK activation generally decreases neuronal firing, leading to neuronal self-inhibition, neuron-to-neuron inhibition and inhibition at the network level [18–20]. In fact, GPCRs-GIRK signaling pathways contribute to many physiological and pathophysiological conditions, such as pain, reward, learning/memory, anxiety, schizophrenia and addiction [21].

GIRK channels have functionally been associated with pain perception, in particular as major mediators for opioid-induced analgesia in both brain and periphery, as evidenced by the use of the specific GIRK channel blocker tertiapin-Q (TTQ) and knock-out (KO) mice [22–26]. For example, GIRK2 channels are critical for the analgesic actions of opioids [22] and GIRK1- and/or GIRK2-KO mice display thermal, mechanical and chemical hyperalgesia [27, 28]. With regard to chronic, or neuropathic pain, which is caused by injury or disease afflicting the nervous system, the understanding of the involvement of GIRKs is limited [29–32]. This contrasts with the comprehensive knowledge of the distribution, function and therapeutic potential of other types of potassium channels, especially voltage gated channels, in peripheral pathways [33].

GIRK subunits are extensively and differentially distributed in the mammalian central nervous system [34–36], but less is known about the distribution of these channels at the spinal level, i.e. in dorsal root ganglia (DRGs) and spinal cord. However, detailed analyses, including immuno-electron microscopy, have been carried out on the mouse spinal dorsal horn [28, 37, 38]. Moreover, based on RT-PCR and electrophysiology, expression of all four subunits of GIRKs in rat DRGs has been reported [39]. Here we examine the native distribution and injury-induced changes in the expression of two GIRK subunits, GIRK1 and -2, in rat DRG neurons and spinal cord using immunohistochemistry (IHC) and in situ hybridization (ISH).

## Results

### Expression of GIRK1 and -2 in control DRGs

**Figure 1:**
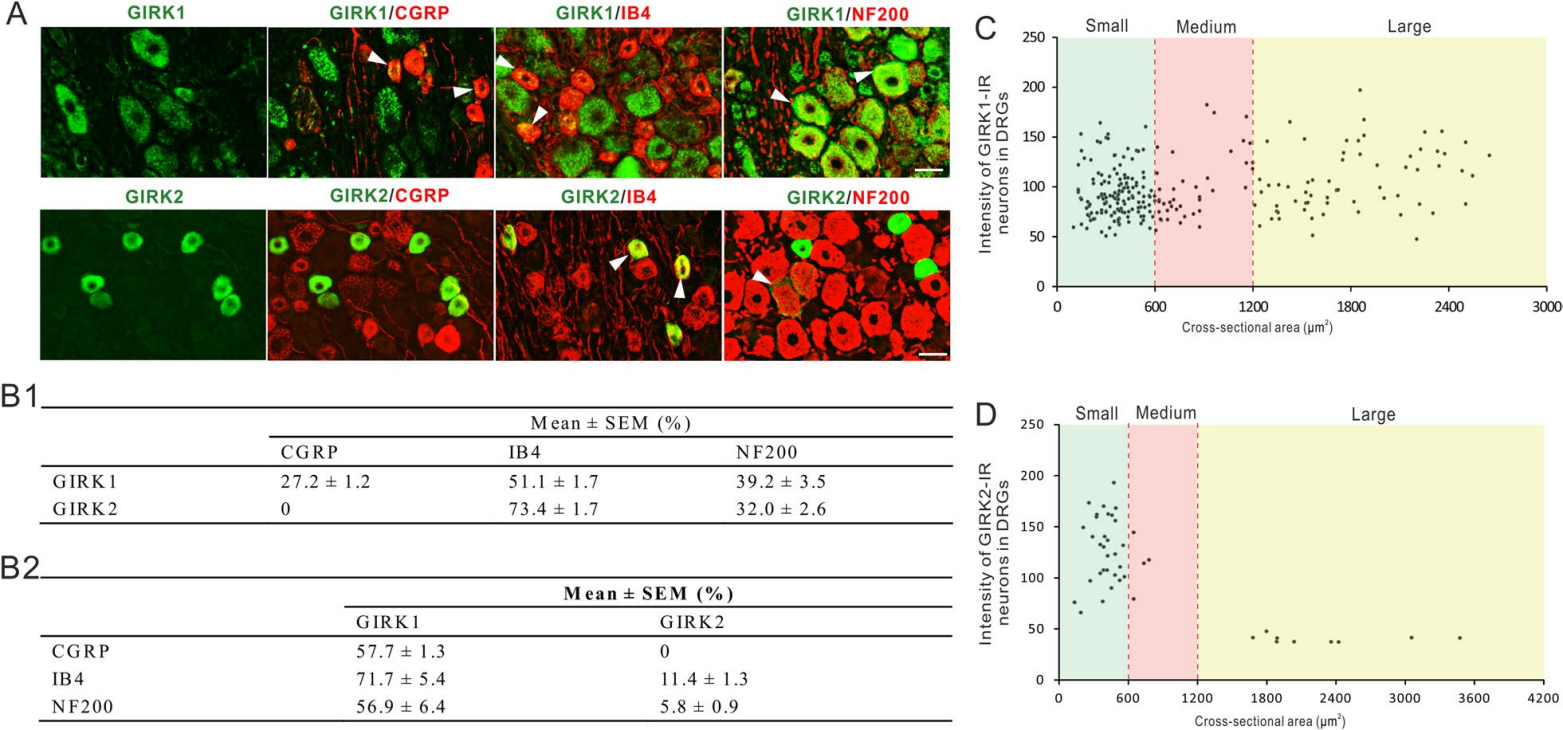
GIRK1 and -2 co-exist with neuronal markers in control DRGs. **A** GIRK1 co-exists with CGRP, IB4 or NF200, whereas GIRK2 only co-exists with IB4 or NF200. *Arrowheads* indicate co-existence of GIRKs with respective markers. **B1** Percentage co-existence of GIRK1^+ ^and -2^+^ NPs with CGRP, IB4 or NF200. **B2** Percentage co-existence of CGRP^+^, IB4^+^ or NF200^+^ NPs with GIRKs. **C** Fluorescence intensity plotted vs. cross-sectional area of GIRK1^+^ neurons. **D** Fluorescence intensity plotted vs. cross-sectional area of GIRK2^+^ neurons. *Scale bar* indicates 40 μm (**A**, valid for all).

**Figure 2:**
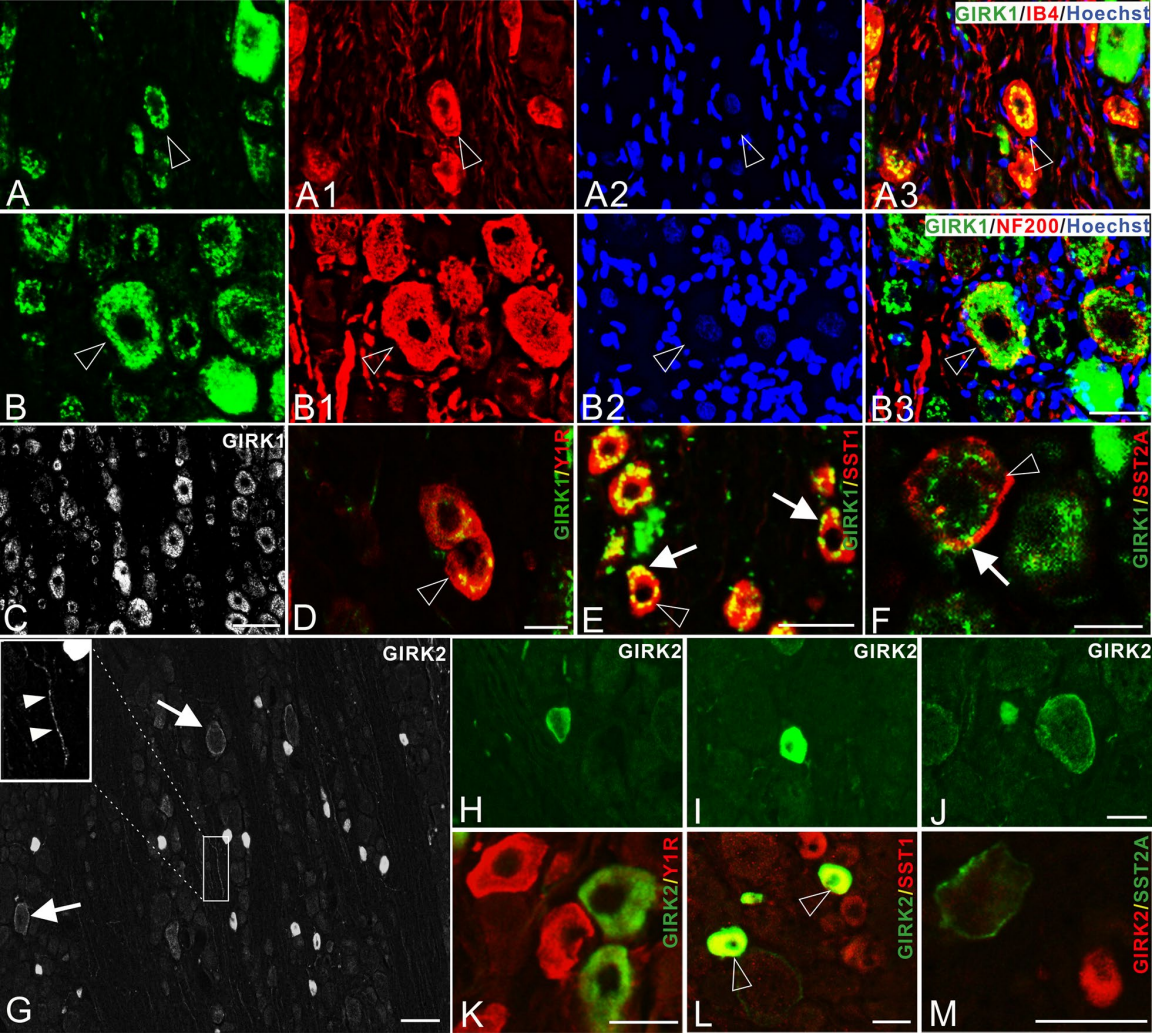
GIRK1 and -2-LIs in control DRG neurons. **A**–**A3** GIRK1 is strongly expressed in the perinuclear region (**A**, **A3**), and IB4-LI in the non-peptidergic DRG neurons (**A1**). Hoechst-LI (**A2**) is a nuclear marker, and *open* *arrowheads* indicate neurons with co-existence (here and below). **B**–**B3** GIRK1-LI is seen throughout the cytoplasm in medium-sized and large neurons (**B**, **B3**), NF200-LI is a marker for large myelinated Aβ fiber neurons (**B1**, **B3**). **C** GIRK1-LI is extensively expressed in DRGs, with varying intensities. **D**–**F** Double-staining shows co-existence of GIRK1 with Y1R (**D**), SST1 (**E**) and SST2A (**F**). *Arrows* point to membrane-association of GIRK1 with the respective GPCR. **G**–**J** GIRK2-LI is found in cell bodies, and also fibers (*boxed area*, *solid arrowheads*) in a DRG (**G**). GIRK2-LI is associated with the cell membrane in both small (**H**) and large neurons (**G**, **J**), and a strong cytoplasmic staining is found in small neurons (**G**, **I**). **K**–**M** Double-staining shows that GIRK2 co-exists with SST1 (**L**), but not with Y1R (**K**) or SST2A (**M**). *Scale bars* indicate 100 μm (**C**, **G**), 40 μm (**A–A3**, **B**–**B3**, **E**, **H**–**J**, **L**, **M**), 20 μm (**D**, **F**, **K**).

Using IHC, GIRK1 and -2 showed different expression patterns in control lumbar 4–5 (L4–5) DRGs (Figures 1, 2). GIRK1 was detected in a large proportion (71.5 ± 1.6%) of DRG neuronal profiles (NPs) of different sizes (Figures 1A, C, 2C), while GIRK2-positive (^+^) NPs were much fewer (8.1 ± 1.1%) and tended to be smaller (Figures 1A, D, 2G). GIRK1- like immunoreactivity (LI) was seen as a granular perinuclear labelling in the cytoplasm of the neurons, and appeared occasionally enriched in the cell membrane (Figure 2A–A3, D–F). GIRK1-LI was predominantly seen throughout the soma of medium-sized and large neurons (Figure 2B–B3). GIRK2 staining was evenly distributed throughout the cytoplasm of small neurons (Figure 2G, I), but was in a few instances also membrane-enriched in small neurons (Figure 2H). In large neurons, GIRK2^+^ staining was always found in cell-membrane compartments (Figures 1A, 2J), which can also be confirmed by the intensity distribution of different sizes of GIRK2^+^ neurons, where all large GIRK2^+^ neurons had a much lower intensity compared with small ones (Figure 1D).

We used calcitonin gene-related peptide (CGRP), isolectin B4 (IB4), and neurofilament 200 (NF200) as phenotypic markers to differentiate small unmyelinated peptidergic, small unmyelinated non-peptidergic and medium-sized and large myelinated neurons, respectively [40]. GIRK1 and GIRK2 showed different distributions among phenotypic characterized neurons. Of all GIRK1^+^ NPs, 27.2 ± 1.2, 51.1 ± 1.7 and 39.2 ± 3.5% co-expressed CGRP, IB4 and NF200, respectively. Conversely, 57.7 ± 1.3, 71.7 ± 5.4, and 56.9 ± 6.4% of the CGRP^+^, IB4^+^, and NF200^+^ NPs expressed GIRK1, respectively (Figure 1A, B). Most GIRK2^+^ NPs contained IB4-reactive glycoprotein (73.4 ± 1.7%) and 32.0 ± 2.6% of GIRK2^+^ NPs expressed NF200, but none CGRP. Conversely, 11.4 ± 1.3 and 5.8 ± 0.9% of IB4^+^ and NF200^+^ NPs expressed GIRK2, respectively (Figure 1A, B).

Previous studies have indicated that GPCR-GIRK modulatory pathways may be involved in abnormal sensations such as neuropathic, inflammatory or arthritic pain [30, 41]. Here, we examined a set of GPCRs that have previously been linked to neuropathic pain, namely neuropeptide Y Y1 receptor (Y1R), somatostatin receptor 1 (SST1) and somatostatin receptor 2A (SST2A), with regard to their co-localization with GIRK1 and -2 in DRGs. Y1R, SST1 and SST2A were, as expected, found on membranes and in the cytoplasm, and all three co-existed with GIRK1, occasionally on the membrane (Figure 2D–F). In GIRK2^+^ neurons, SST1-LI, but not SST2A-LI or Y1R-LI, was observed (Figure 2K–M).

**Figure 3:**
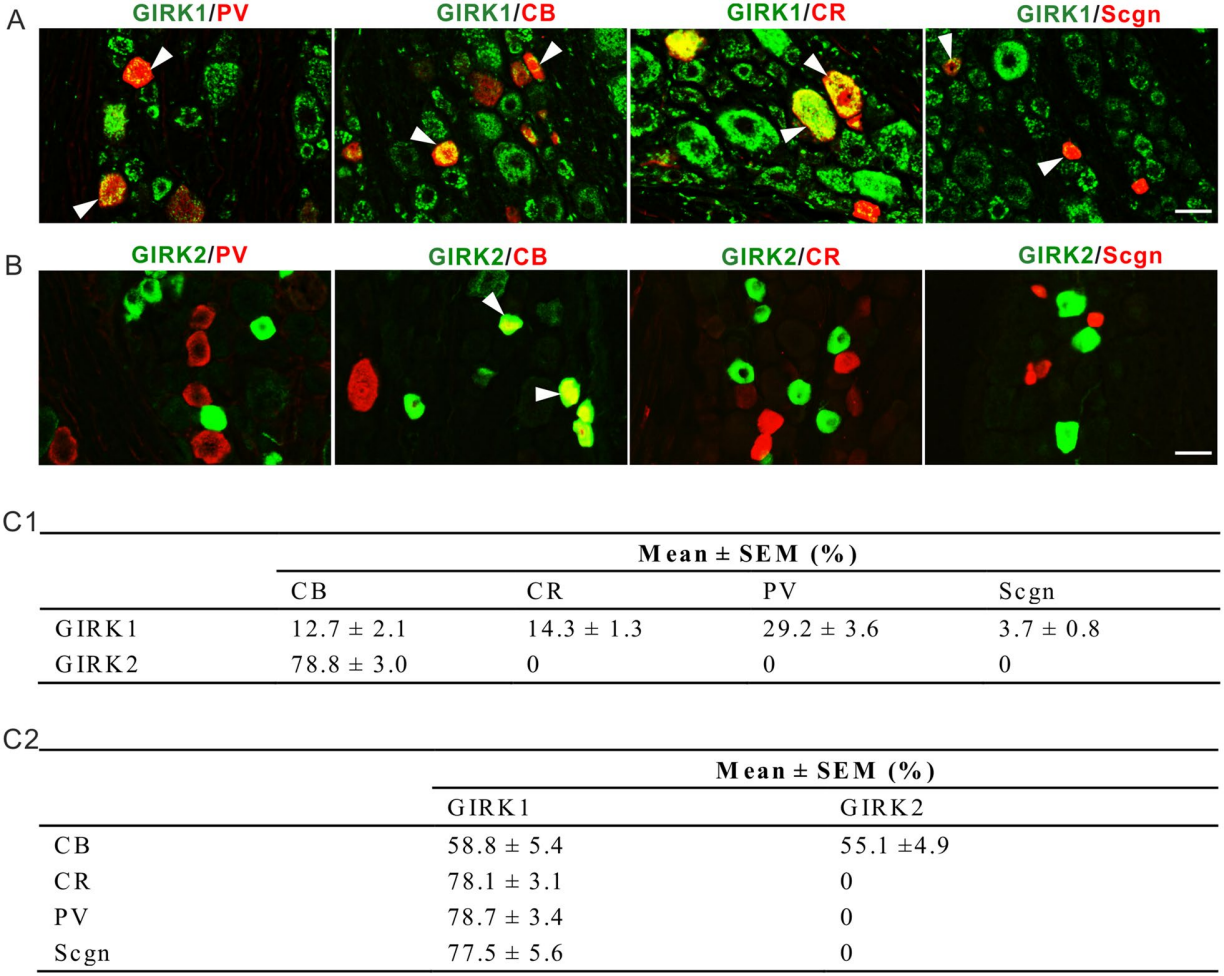
GIRK1 and -2 co-exist with CaBPs in control DRG neurons. **A** GIRK1 co-exists with PV, CB, CR or Scgn. *Arrowheads* indicate co-existence of GIRK1 with the respective CaBP (*yellow*). **B** GIRK2 co-exists with PV, CB, CR or Scgn. *Arrowheads* indicate co-existence of GIRK2 with CB (*yellow*). This co-existence is only found in small neurons. **C** Quantification analysis shows the percentage of GIRK1^+^ and -2^+^ NPs that co-express the respective CaBPs (**C1**), and conversely the percentage of the respective CaBP^+^ NPs that co-expresses GIRK1 or -2 (**C2**). *Scale bars* indicate 40 μm (**A**, **B**, valid for all).

To further characterize the distribution of GIRK1 and GIRK2 among DRG neurons, four calcium-binding proteins (CaBPs), calbindin D28k (CB), calretinin (CR), parvalbumin (PV) and secretagogin (Scgn), were used as markers [42–46]. We found that 12.7 ± 2.1, 14.3 ± 1.3, 29.2 ± 3.6 and 3.7 ± 0.8% of the GIRK1^+^ NPs expressed CB, CR, PV and Scgn, respectively. Conversely, 58.8 ± 5.4, 78.1 ± 3.1, 78.7 ± 3.4 and 77.5 ± 5.6% of the CB^+^, CR^+^, PV^+^ and Scgn^+^ NPs expressed GIRK1, respectively (Figure 3A, C). Moreover, 78.8 ± 3.0% of the GIRK2^+^ NPs expressed CB, but none expressed any of the other three CaBPs. 55.1 ± 4.9% of the CB^+^ NPs expressed GIRK2 (Figure 3B, C).

Thus, GIRK1 was more diversified in its co-localization with CaBPs as compared to GIRK2, which only expressed one (CB) of the four tested CaBPs.

### Co-expression of GIRK1 and -2 with galanin and nNOS in DRGs after axotomy

**Figure 4:**
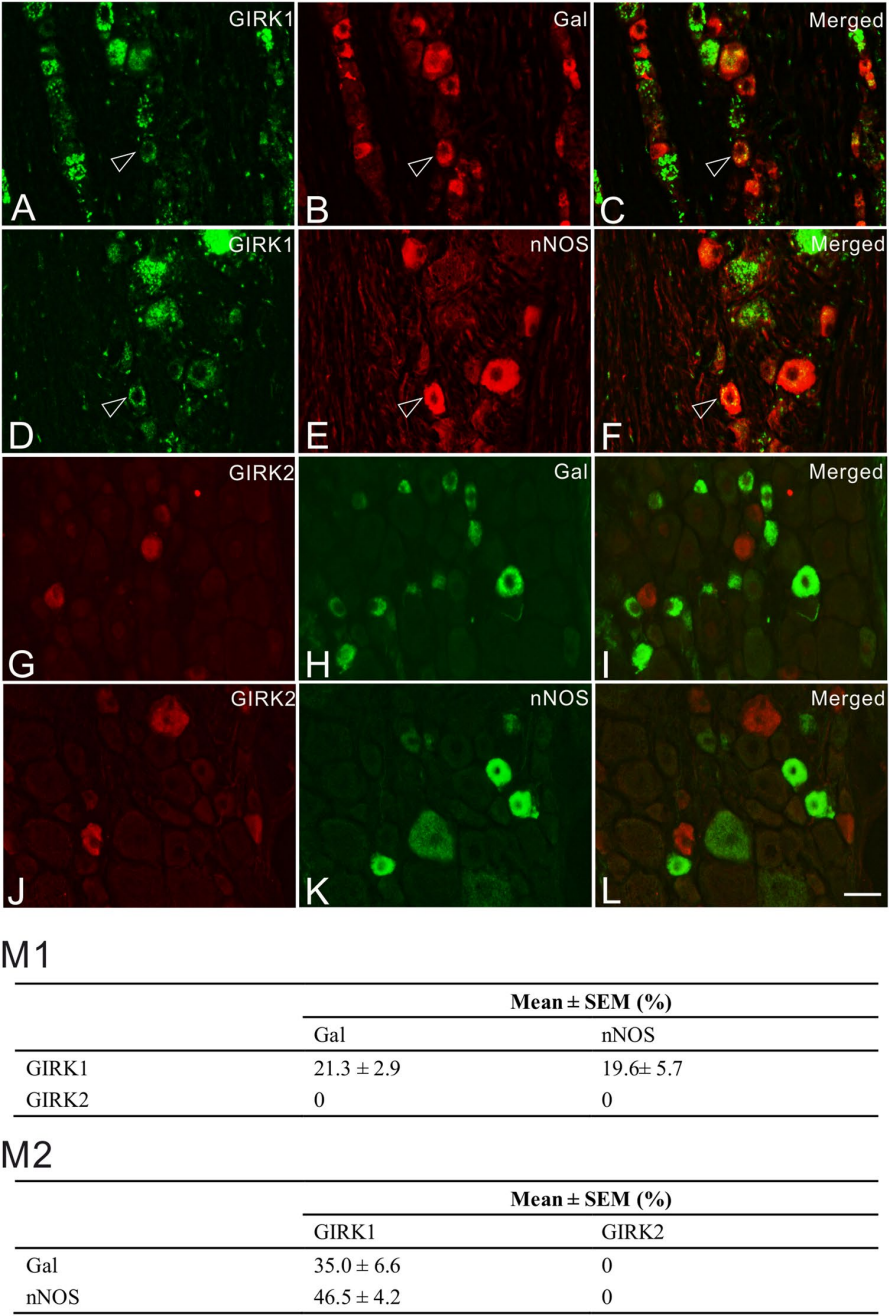
GIRK1 and -2 co-exist with galanin (Gal) or nNOS in ipsilateral DRG neurons after unilateral axotomy. **A**–**F** Double staining shows that GIRK1 (**A**, **D**) co-exists with Gal (**B**, **C**) and nNOS (**E**, **F**). *Arrowheads* indicate co-existence (*yellow*). **G**–**L** Double staining shows that GIRK2 (**G**, **J**) neither co-exists with Gal (**H**, ** I**) nor nNOS (**K**, ** L**). **M** Quantification analysis shows the percentage of GIRK1^+^ and -2^+^ NPs that co-express Gal or nNOS (**M1**), and conversely the percentage of Gal^+^ and nNOS^+^ NPs that co-express GIRK1 or -2 (**M2**). *Scale bar* indicates 40 μm (**A**–**L**, valid for all).

Altered expression of molecular components of signaling pathways in DRG neurons induced by peripheral nerve injury is usually regarded as one of the mechanisms for long-term modulation underpinning neuropathic pain development [47]. Two biomarkers, galanin [48] and nNOS [49, 50], have been shown to be regulated in ipsilateral rat DRGs after peripheral nerve injury and are suggested to be involved in chronic pain states. Here we show that GIRK1, but not GIRK2, co-localized with both these molecular markers (Figure 4). We found 21.3 ± 2.9 and 19.6 ± 5.7% of GIRK1^+^ NPs expressed galanin and nNOS, respectively. Conversely, 35.0 ± 6.6, 46.5 ± 4.2% of the galanin^+^ and nNOS^+^ expressed GIRK1, respectively (Figure 4M).

### Expression of GIRK1 and -2 in spinal cord

**Figure 5:**
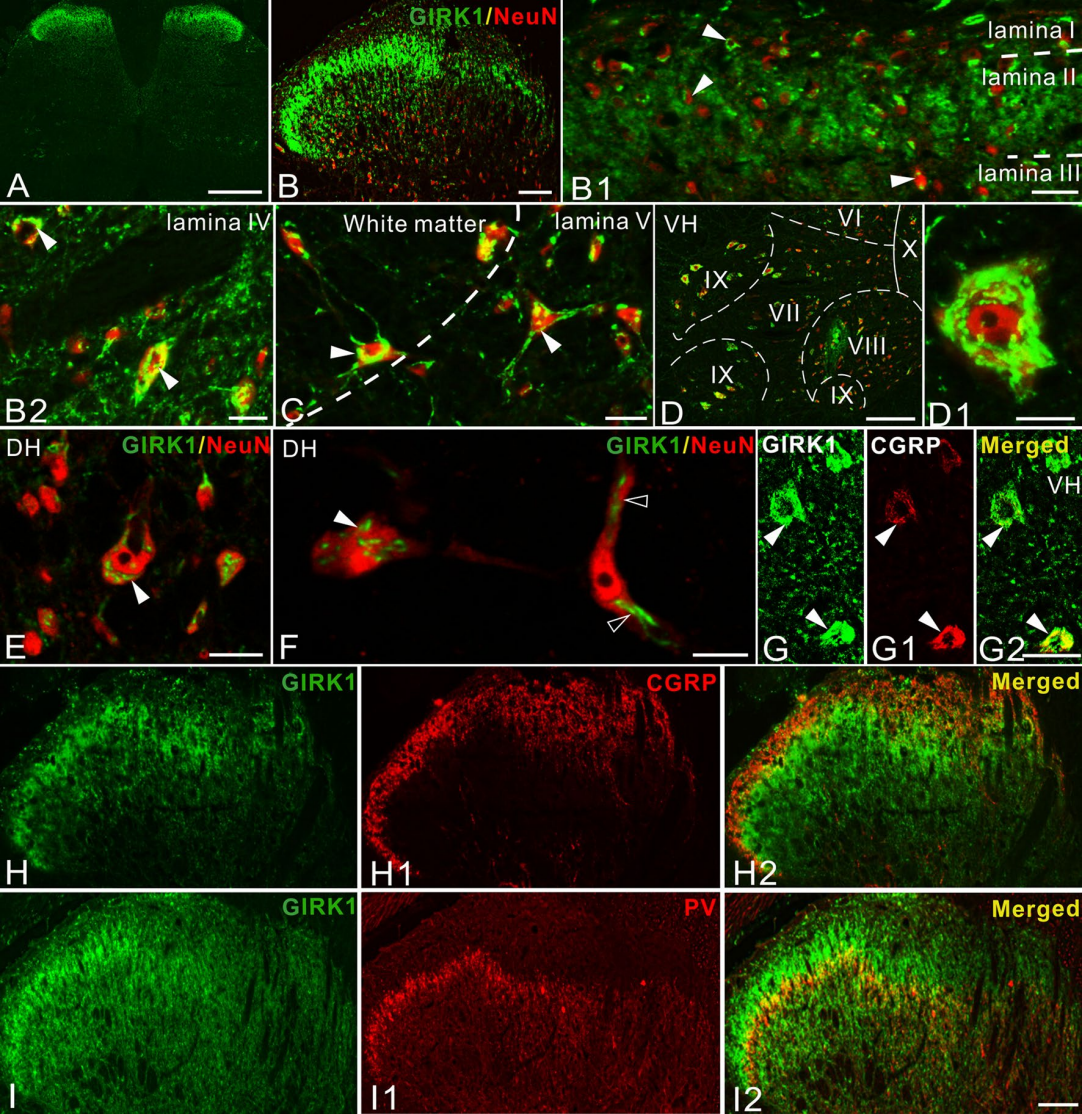
GIRK1-LI in L4-5 segments of control spinal cord. **A** GIRK1-LI in the L4-5 spinal cord at low magnification. **B** GIRK1-LI in the dorsal horn (DH). GIRK1-IR processes are mainly present in superficial layers. NeuN-LI (here and below) is used to identify neurons. **B1**–**B2** Double-staining shows the distribution of GIRK1-IR cell bodies in different DH laminae, from the superficial to deep layers. **C** GIRK1-IR multipolar neurons are seen both in white matter and deep DH layers at the white matter border. **D** GIRK1 is extensively expressed in ventral horn (VH) neurons, in lamina VI to IX, and around the central canal (lamina X). **D1** High magnification image shows the typical expression pattern of GIRK1-LI in a VH neuron. **E**, **F** A few GIRK1^+^ neurons in DH show dot-like immunoreactivity in soma (*solid arrowheads*) and processes (*open arrowheads*). (**G**–**G2**) CGRP^+^ motor neurons (**G1**) express GIRK1 (**G**). (**H**–**H2**) GIRK1-LI (**H**, **H2**) and CGRP-LI (**H1**, **H2**) show only limited overlap. (**I**–**I2**) GIRK1-LI (**I**) overlaps with PV-LI (**I1**) in inner lamina II (**I2**). *Scale bars* indicate 500 μm (**A**), 200 μm (**D**, **G**–**G2**), 100 μm (**B**, **H**–**I2**), 20 μm (**B1**, **B2**, **C**, **D1**, **E**), 10 μm (**F**).

**Figure 6:**
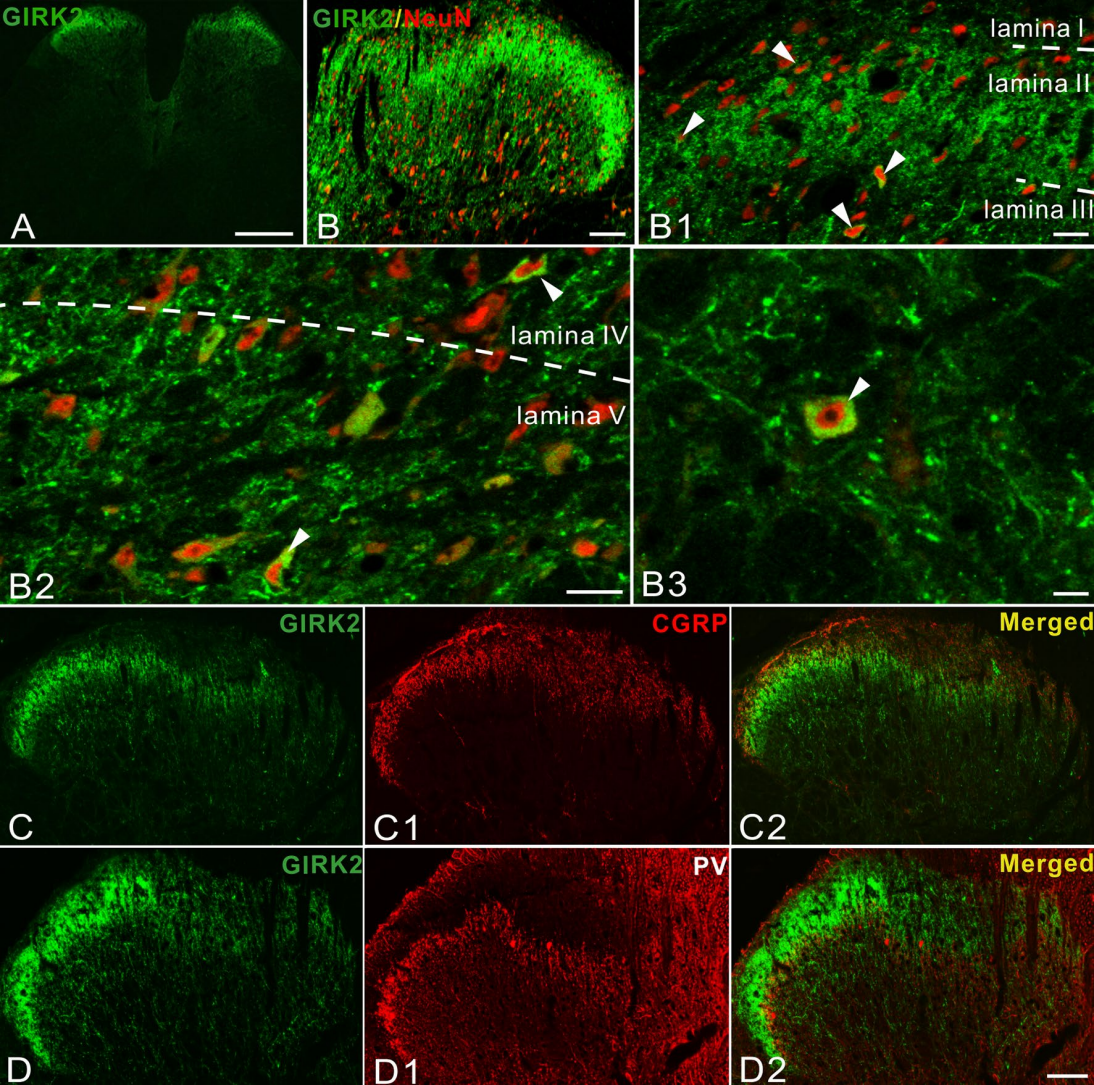
GIRK2-LI in L4-5 segments of control spinal cord. **A** GIRK2-LI in the L4-5 spinal cord at low magnification. **(B**–**B2)** Double-staining shows GIRK2-IR neuron distribution in different dorsal horn laminae, from the superficial to the deeper layers. NeuN-LI is used to identify neurons (here and below). (**B3**) Typical cytoplasmic staining pattern of GIRK2-LI. (**C**–**C2**) GIRK2- (**C**) and CGRP-LIs (**C1**) partly overlap in the dorsal horn (**C2**). (**D–D2**) GIRK2-LI (**D**) overlaps with PV-LI (**D1**) in inner lamina II (**D2**). *Scale bars* indicate 500 μm (**A**), 100 μm (**B**, **C**–**D2**), 20 μm (**B1**, **B2**), 10 μm (**B3**).

Dense networks of GIRK1^+^ and -2^+^ nerve terminals and neuronal cell bodies were observed in the superficial layers of the spinal dorsal horn (Figures 5A, B, 6A, B). GIRK1^+^ and GIRK2^+^ fibers were most abundant in the lateral aspects and presented in outer and inner lamina II, as well as at the border between lamina II and III (Figures 5H, H2, I, I2, 6C, C2, D, D2). GIRK1- and -2-immunoreactive (IR) neuronal cell bodies, including multipolar neurons, were seen in different layers (Figures 5 B1–C, E, F, 6B1–B3). Some multipolar GIRK1^+^, but no GIRK2^+^, interneurons were found in the border area between grey and white matter at the level of lamina V (Figure 5C).

In the ventral horn numerous GIRK1- IR neurons were observed, extending from lamina VI to IX, also including lamina X surrounding the central canal (Figure 5D, D1). All CGRP^+^ motoneurons expressed GIRK1 (Figure 5G–G2). GIRK2-IR neurons were not detected in the ventral spinal cord.

**Figure 7:**
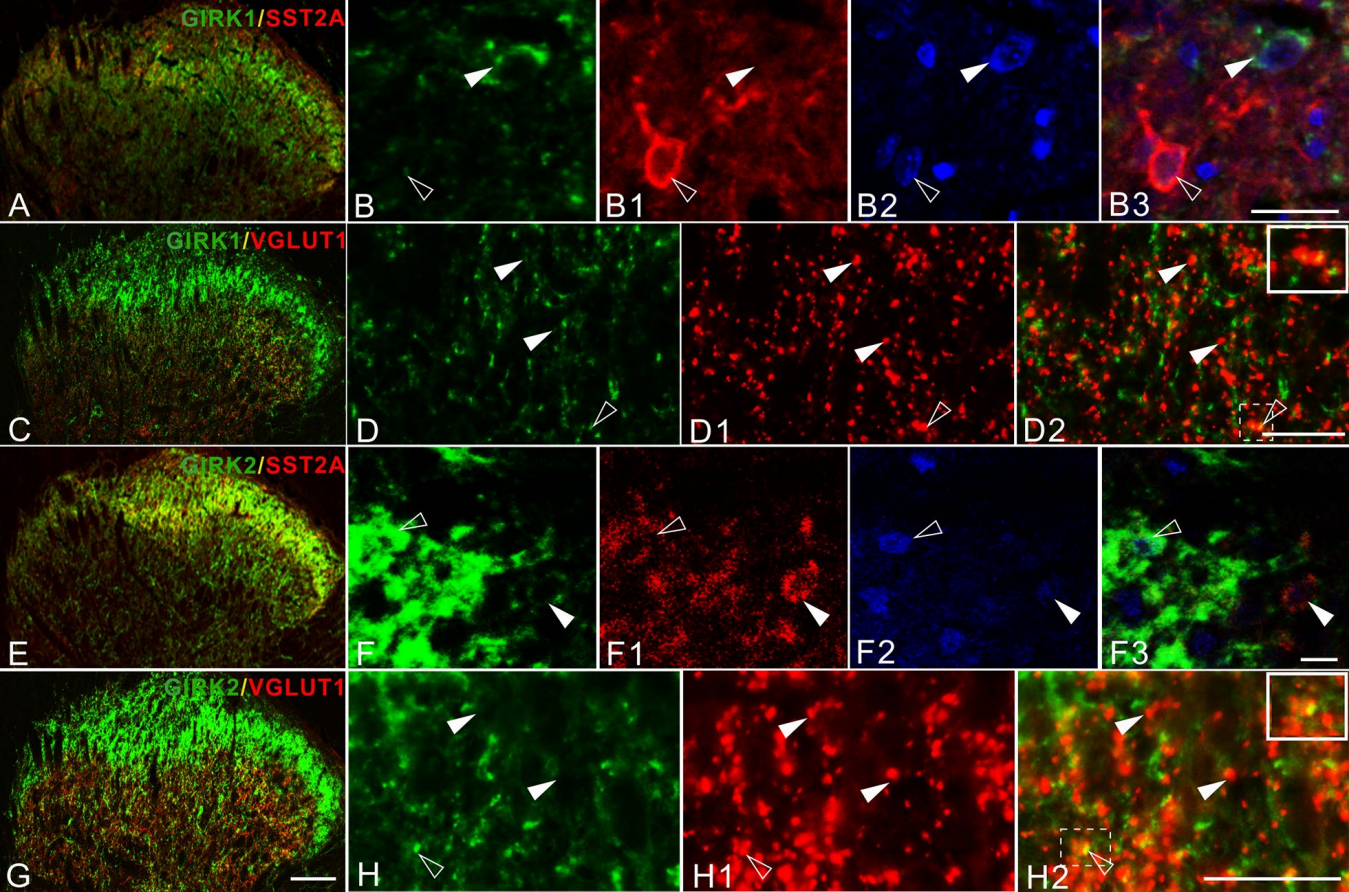
The two GIRKs overlap with SST2A and VGLUT1 in control spinal dorsal horn. **A** GIRK1-LI overlaps with SST2A-LI. **B**–**B3** Triple-staining (**B3**) for GIRK1 (**B**), SST2A (**B1**) and Hoechst (**B2**) shows no co-existence of GIRK1-LI (*solid arrowheads*) with SST2A-LI (*open arrowheads*) in the superficial laminae. **C** GIRK1-LI overlaps with VGLUT1-LI in the superficial laminae. **D–D2** Double-staining (**D2**) shows that only a few VGLUT1-IR boutons (**D2**) are GIRK1^+^ (**D**, **D2**) (*open arrowheads*), as indicated in the high power magnification (*upper box*, **D2**). Most of VGLUT1^+^ boutons have no GIKR1-LI (*solid arrowheads*). **E** GIRK2-LI overlaps with SST2A-LI in superficial laminae. **F**–**F3** Triple-staining (**F3**) for GIRK2 (**F**), SST2A (**F1**) and Hoechst (**F2**) shows a possible membrane associated co-localization of GIRK2 and SST2A (*solid arrowheads*), *open arrowheads* show the GIRK2^+^, but SST2A-negative (^−^) neurons. (**G**) GIRK2-LI overlaps with VGLUT1-LI. (**H**–**H2**) Double-staining (**H2**) for GIRK2 (**H**) and VGLUT1 (**H1**) shows that some VGLUT1^+^ boutons are GIRK2^+^ in the spinal dorsal horn (*open arrowheads*), as shown in high power magnification (*upper box*, **H2**). Most of VGLUT1^+^ boutons are however GIRK2^−^ (*solid arrowheads*). *Scale bars* indicate 100 μm (**A**, **C**, **E**, **G**), 25 μm (**D**–**D2**, **H**–**H2**), 20 μm (**B**–**B3**), 10 μm (**F**–**F3**).

In order to further investigate the phenotype of GIRK1^+^ and GIRK2^+^ neurons in the spinal dorsal horn, SST2A was used as a marker for GABAergic inhibitory interneurons in lamina I–II. The SST2A^+^ interneurons represent >50% of all neurons in this region [51, 52]. We did not observe any co-existence of GIRK1 with SST2A, while occasional examples of neurons co-expressing GIRK2 and SST2A were found (Figure 7A–B3, E–F3). To identify excitatory nerve terminals in dorsal horn, we used vesicle glutamate transporter 1 (VGLUT1) as a marker. Some examples of co-localization between GIRK1- or -2-LI with VGLUT1-LI were noted (Figure 7C–D2, G–H2).

### Expression of GIRK1 and -2 proteins and mRNAs in DRGs and dorsal horn after axotomy

**Figure 8:**
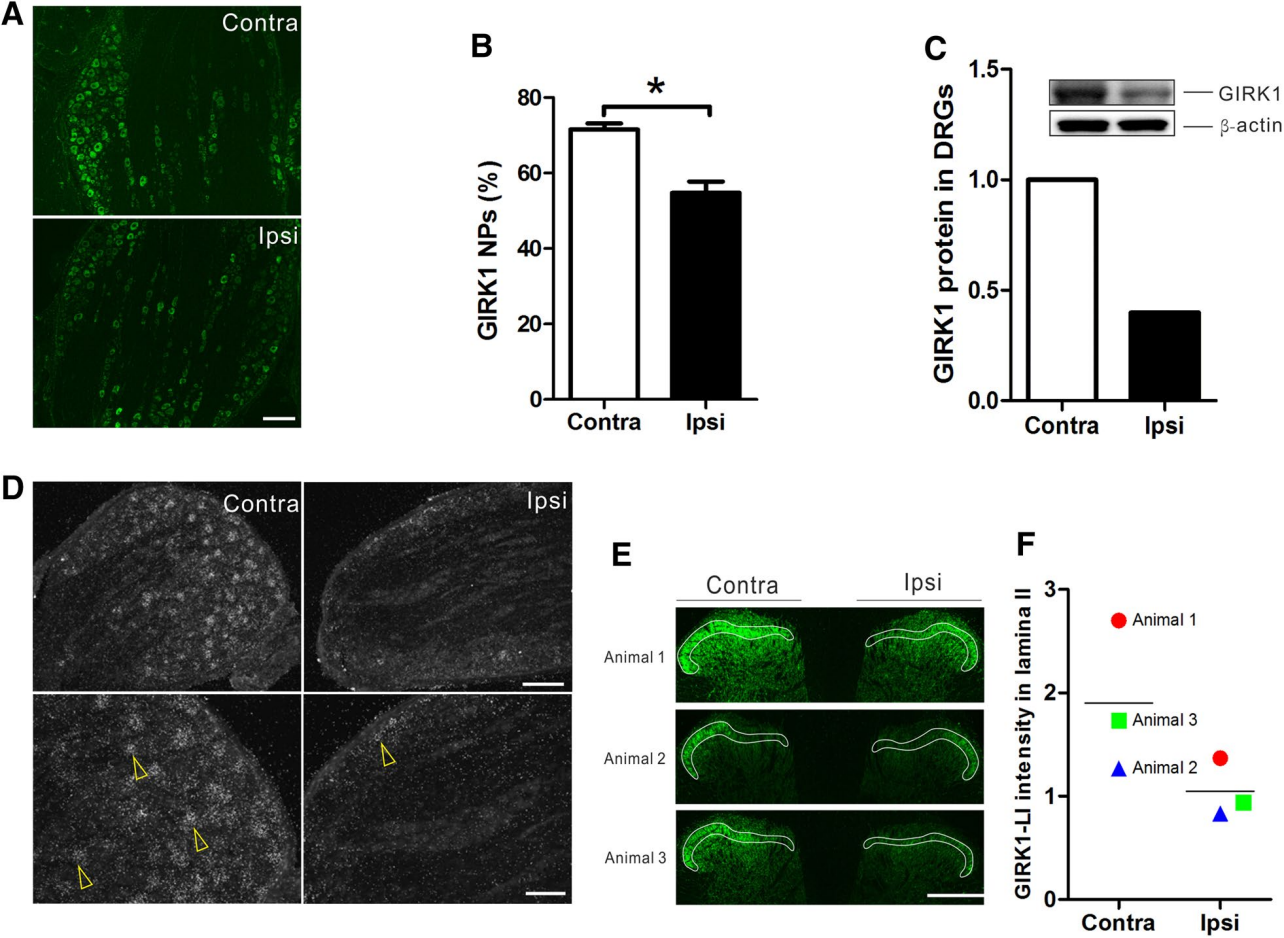
Protein and mRNA expression of GIRK1 in DRGs and spinal cord 14 days after axotomy. **A**, **B** Percentage of GIRK1^+^ NPs is significantly decreased in the ipsilateral (n = 8) as compared to contralateral DRGs (n = 8). **C** Western blot result showing GIRK1 protein levels confirms this effect (n = 4 per group). **D** GIRK1 mRNA is extensively distributed in the contralateral DRG and is ipsilaterally down-regulated after axotomy (*upper panel*: low magnification; *lower panel*: high magnification). **E** GIRK1-LI (within *white frame*) in the dorsal horn. **F** Monitoring fluorescence intensity in the dorsal horn shows down-regulation of GIRK1-LI in all animals (n = 3 per group). *P < 0.05. *Scale bars* indicate 500 μm (**E**), 200 μm (**D**, *upper panel*; **A**) and 100 μm (**D**, *lower panel*).

**Figure 9:**
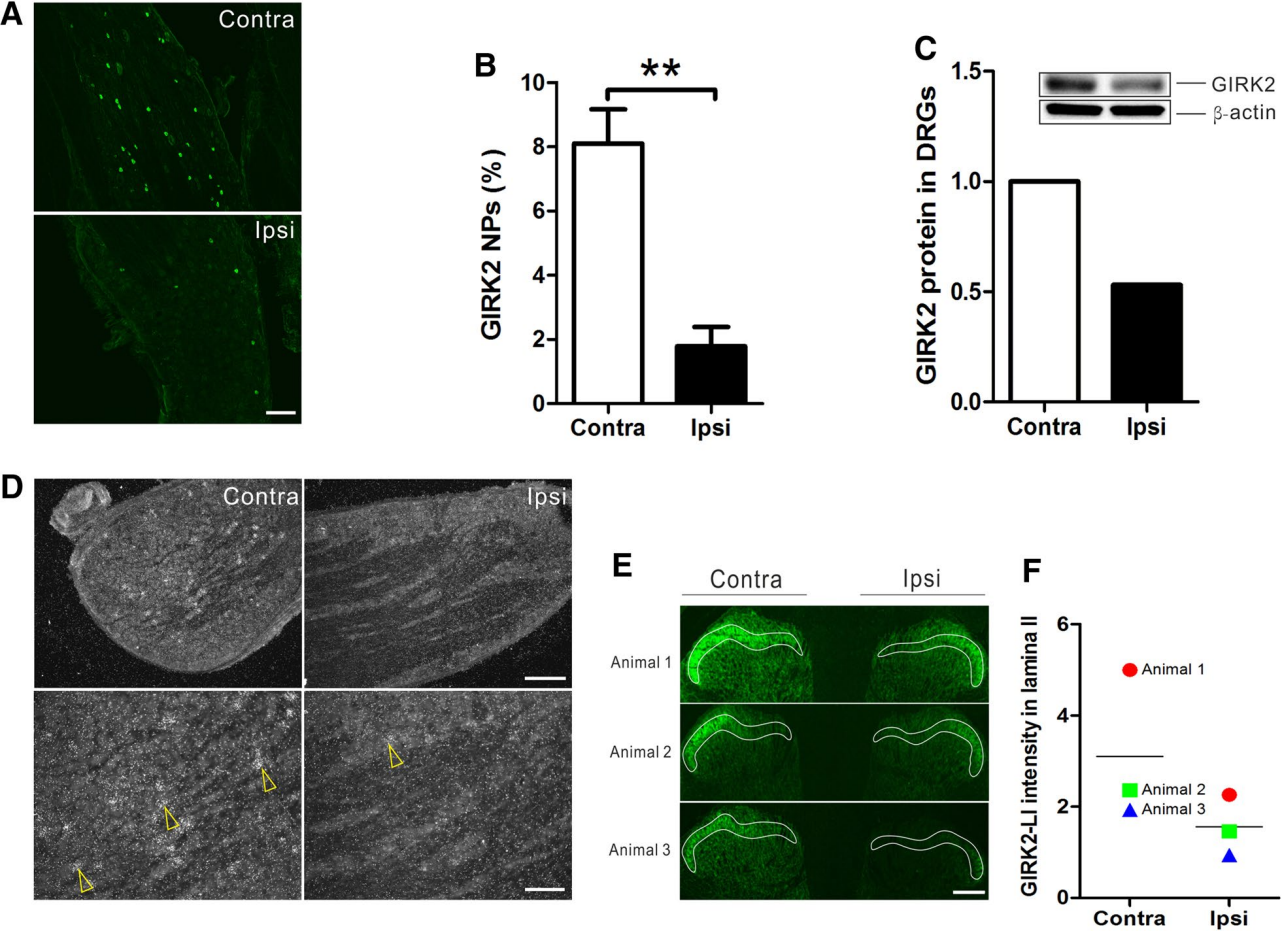
Protein and mRNA expression of GIRK2 in DRGs and spinal cord 14 days after axotomy. **A**, **B** Percentages of GIRK2^+^ NPs is significantly decreased in the ipsilateral (n = 8) as compared to the contralateral DRGs (n = 8). **C** Western blot result showing GIRK2 protein levels confirms this effect (n = 4 per group). **D** GIRK2 mRNA shows a modest signal in the contralateral DRG, and is down-regulated in the ipsilateral DRG after axotomy (*upper panel*: low magnification; *lower panel*: high magnification). **E** GIRK2-LI (within *white frame*) in the dorsal horn. **F** Monitoring fluorescence intensity in the dorsal horn shows down-regulation of GIRK2-LI in all animals (n = 3 per group). **P < 0.01. *Scale bars* indicate 200 μm (**D**, *upper panel*; **A** and **E**), 100 μm (**D**, *lower panel*).

Fourteen days after axotomy, a significant decrease in the percentage of GIRK1-IR and GIRK2-IR NPs was observed in the ipsilateral as compared to contralateral DRGs (GIRK1, 54.7 ± 3.0 vs. 71.53 ± 1.6%, p < 0.05; GIRK2, 1.8 ± 0.6 vs. 8.1 ± 1.1%, p < 0.01, n = 8 per group; Figures 8A, B, 9A, B). The surgical denervation of the DRGs was confirmed by an up-regulation of galanin-LI in NPs of the ipsilateral DRGs (data not shown) [53]. We next used western blot to detect total levels of GIRK1 and -2 proteins in contra- and ipsilateral DRGs after nerve injury. Our results confirmed that both GIRK1 and -2 subunits were down-regulated by axotomy (Figures 8C, 9C).

In situ hybridization demonstrated moderate GIRK1 mRNA levels in a large proportion of neurons in contralateral DRGs, while the GIRK2 mRNA signal was restricted and weak. In ipsilateral DRGs, however, a strong reduction of GIRK1 and GIRK2 mRNA levels was evident as compared to the contralateral DRGs (Figures 8D, 9D), respectively. Taken together, the data indicated a marked down-regulation of both protein and mRNA levels of GIRK1 and -2 in DRGs after peripheral nerve injury.

Peripheral nerve injury also induced change in protein expression in the dorsal horn. Thus, 14 days after axotomy we detected a strong reduction in GIRK1 and -2-LIs in the ipsilateral dorsal horn lamina II (Figures 8E, F, 9E, F).

### Axonal transport of GIRK1 and -2

**Figure 10:**
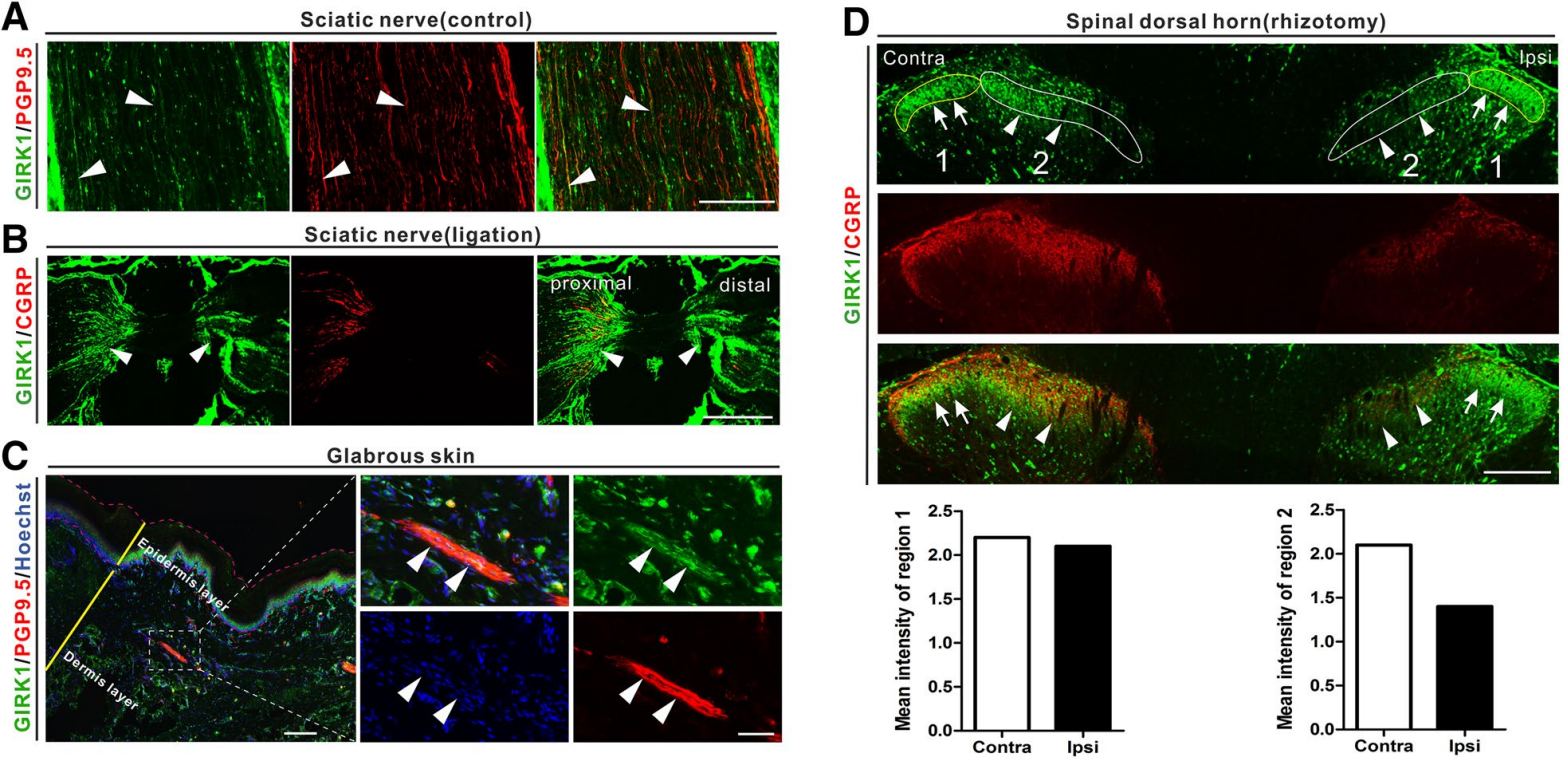
Axonal transport of GIRK1-LI. **A** GIRK1-LI is observed in many PGP9.5^+^ fibers in intact sciatic nerve. **B** After a 10-h nerve ligation, a strong accumulation of GIRK1-LI is seen on both proximal and distal side (*arrowheads*). **C** A few GIRK1^+^ and PGP9.5^+^ fibers (*arrowheads*) are observed in the epidermis layer of glabrous skin in rat hind paw. **D** Twenty-one days after unilateral lumbar dorsal rhizotomy, a reduction of GIRK1-LI is seen within region 2 (*arrowheads*), but not in region 1 (*arrows*), as compared with the contralateral side. A dramatic reduction of CGRP-LI is seen ipsilaterally. *Scale bars* indicate 500 μm (**B**), 200 μm (**A**, **C**, low magnification; **D**), 50 μm (**C**, high magnification).

**Figure 11:**
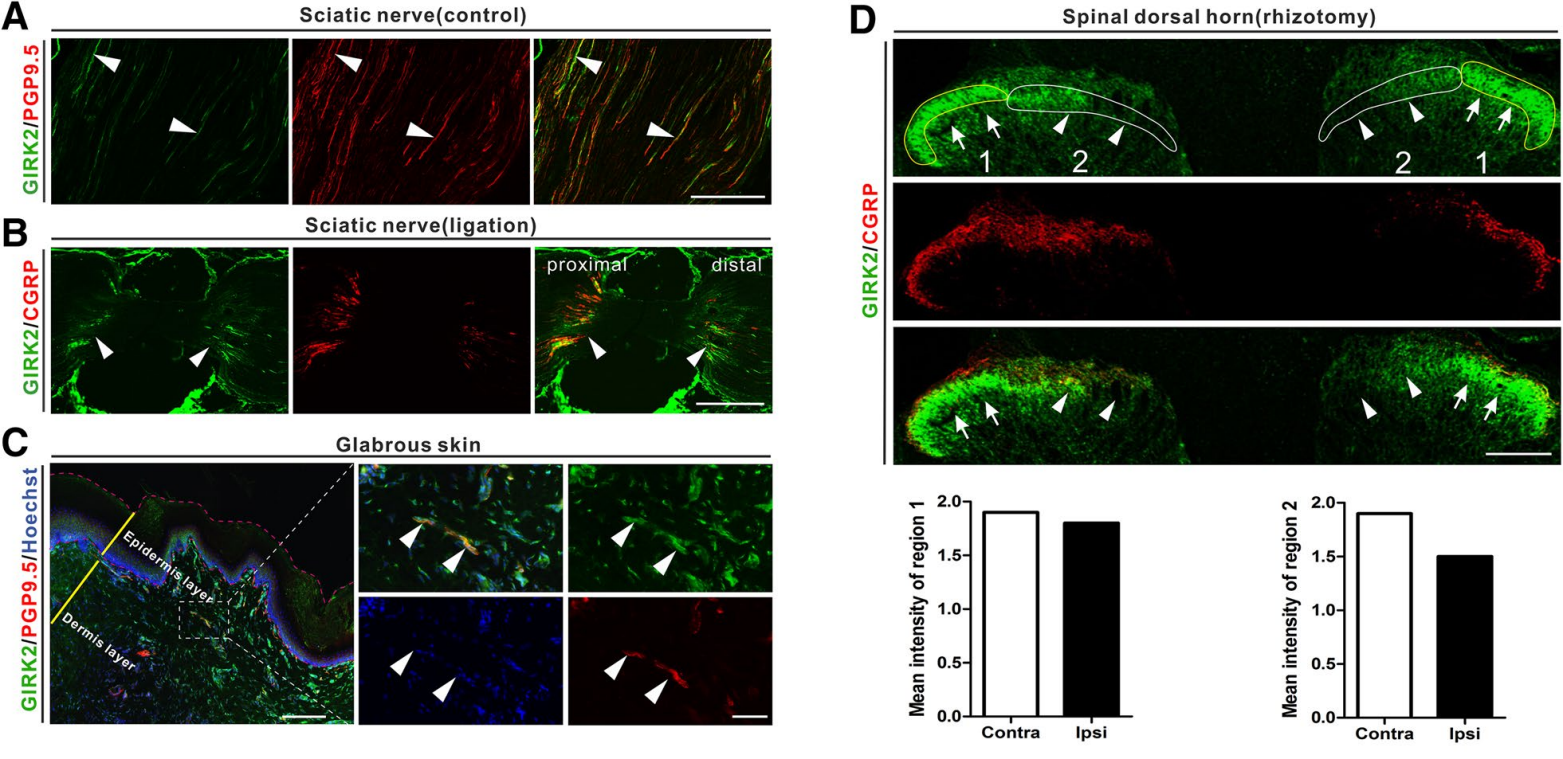
Axonal transport of GIRK2-LI. **A** GIRK2-LI is observed in many PGP9.5^+^ fibers in intact sciatic nerve. **B** After a 10-h nerve ligation, a strong accumulation of GIRK2-LI is seen on both proximal and distal side (*arrowheads*). **C** A few GIRK2^+^ and PGP9.5^+^ fibers (*arrowheads*) are observed in the epidermis layer of glabrous skin in rat hind paw. **D** Twenty-one days after unilateral lumbar dorsal rhizotomy, a reduction of GIRK2-LI is seen within region 2 (*arrowheads*), but not in region 1 (*arrows*), as compared with the contralateral side. A strong reduction of CGRP-LI is seen ipsilaterally. *Scale bars* indicate 500 μm (**B**), 200 μm (**A**, **C**, low magnification; **D**), 50 μm (**C**, high magnification).

GIRK1- and -2-LIs were observed in nerve fibers of the intact sciatic nerve. Double-labelling with the axonal marker PGP9.5 suggested co-localization with GIRKs in control nerve and after ligation. This was especially distinct with GIRK2 (Figures 10A, 11A). After sciatic nerve ligation, GIRK1-LI and -2-LIs accumulated both proximal and distal to the ligation (Figures 10B, 11B). We also detected GIRK1^+^ and GIRK2^+^ nerve fibers in dermis layers of glabrous skin of the hind paw (Figures 10C, 11C). Dorsal rhizotomy markedly reduced GIRK1-LI in the mid/lateral region in lamina II of the dorsal horn (Figure 10D). A similar but less pronounced effect was seen for GIRK2-LI (Figure 11D).

## Discussion

In the present study we report that GIRK1 is expressed in a majority of DRG neurons of different sizes and thus associated with different modalities, whereas GIRK2 is limited to a small subpopulation of nociceptors. Both are down-regulated after peripheral nerve injury. These results provide histochemical support for involvement of GIRKs in pain processing at the spinal level as reported in earlier studies [29, 30, 38], and complement a large number of studies showing a similar regulation, and presumable function, of voltage gated K^+^ channels, which also are down-regulated by peripheral nerve injury [31, 33, 54–62].

### GIRK1 and -2 mRNAs in DRGs

In the current study, we focused on the expression of GIRK1 and -2 in DRGs and spinal cord under native and neuropathic pain conditions. Our qPCR and ISH results show that GIRK1 and -2 mRNAs are expressed in rat DRGs, which is in agreement with a previous study on rat based solely on RT-PCR [39]. The GIRK1 and -2 mRNA signals were robust (Ct values around 24 and 28, respectively, data not shown). Also with ISH we could detect transcripts for GIRK1 and -2, both of which undergo alternative splicing [3, 4].

### GIRK1 and -2 proteins in DRGs

So far, little is known about the neurochemical phenotype of GIRK1 and -2-IR neurons in rat DRGs and spinal cord. Here we observed a patchy, cytoplasmic and perinuclear GIRK1-IR staining around the nucleus, suggestive of endoplasmic reticulum (ER) localization, while a distinct association with the neuronal cell membrane was rarely observed. This fits with the notion that GIRK1 lacks an ER export signal, and must associate with another GIRK subunit for expression in the plasma membrane [8, 63]. Here GIRK2 would be suitable, which was extensively expressed in both cytoplasm and on membranes, however in a fairly small population. Almost 73% of the GIRK2^+^ and ~50% of the GIRK1^+^ neurons were IB4^+^, indicating that nociceptors might express GIRK1/2 heterotetrameric channels. In contrast, around 27% of the GIRK1^+^, but no GIRK2^+^, neurons expressed CGRP; thus it is unlikely that heterotetramers between these two GIRKs exist in peptidergic nociceptors [40].

The large myelinated neurons, identified by NF200, give rise to A-fibers, including Aβ fibres, and mostly subserve mechansensory functions [64]. In this cell population, GIRK1-LI was expressed in ~39% of the NPs throughout the cytoplasm. GIRK2-LI was found in around one-third of these neurons, and the immunoreactivity was always associated with the cell membrane. Thus, in these neurons functional GIRK1/2 heterotetramers may exist.

Further phenotyping showed that different small GIRK1^+^ neuron subpopulations express all four CaBPs studied, the largest one expressing PV (~30%). GIRK2^+^ neurons only expressed CB. The functional implications of these results remain to be resolved.

### GIRK1 and -2 interaction with inhibitory GPCRs

G protein-induced opening of GIRK channels typically results in a several-fold increase in potassium conductance at the resting membrane potential. GIRK opening is one of the fastest metabotropic effector mechanisms, owing to its short signaling pathway, with the G protein as the single link between the receptor and the channel. As such, it well suited for rapid responses to neuromodulators [1].

Double immunofluorescence staining of DRG neurons showed that GIRK1 co-exists with three neuropeptide receptors linked to inhibitory neurotransmission and neuropathic pain: the Y1, SST1 and SST2A receptors [65]. All these receptors showed a clear membrane association, and in some cases an overlap with GIRK1 staining was seen, possibly indicating functional interaction. GIRK2 was only associated with SST1^+^ neurons.

GIRK channels are downstream effectors of SST4 for its analgesic effects in rat DRGs [16, 66], and somatostatin may induce slow inhibitory, postsynaptic currents (IPSCs) through activation of GIRK channels [12]. However, it has been reported that the SST1 receptor, in contrast to SST2-5, does not open GIRK channels but might instead decrease their currents [67, 68]. Therefore, somatostatin may play different roles in different neuronal populations in DRGs.

Furthermore, NPY may via Y1 receptors, and GIRK channels cause postsynaptic hyperpolarization in tonic firing neurons in substantia gelatinosa [69]. Also in other regions of the central nervous system NPY acts via GIRK channels, e.g., inhibitory effects of NPY in lateral amygdala have been shown to be mediated through Y1 receptor and GIRK1, 2, and 3 [70].

Also for galanin actions GIRK channels are important, since the anticonvulsant effect of a GalR1 agonist was abolished by the GIRK channel inhibitor tertiapin Q [71]. In addition, galanin has been associated with GIRKs at the spinal level [72]. Unfortunately there is no reliable antiserum recognizing any of the three galanin receptors, GalR1-3, but both GalR1 and GalR3 mediated inhibition via opening of potassium channels [17, 73]. ISH has shown that >40% of the small and medium-sized NPs in rat DRGs express GalR1 mRNA (and ~75% GalR2) [74].

GIRKs have mainly been associated with postsynaptic receptors (see below), but DRG neuron cell bodies are not considered to be ‘innervated’ by nerve endings and thus do not seem to represent a postsynaptic structure. However, it was previously shown that the neuropeptide substance P can be released from DRG neuron cell soma [75], and thus influences adjacent ‘postsynaptic’ DRG cell bodies in a paracrine way, or acts on the releasing cell itself (‘autocrine’ signaling). We have proposed that also NPY and galanin, especially after nerve injury-induced up-regulation, in a similar fashion can be released from DRG cell soma [76], and thus be involved in chemically mediated cross-excitation in DRGs as proposed by Amir and Devor [77]. Here, GIRKs expressed at the DRG neuron cell membrane may be involved in NPY Y1 and GalR1 signaling.

### GIRK1 and -2 in spinal cord

In lamina II of mouse dorsal spinal horn, GIRK1- and -2-LI have previously been detected in excitatory interneurons (“almost exclusively in postsynaptic membranes”) by immunoelectron microscopy [28, 37, 38]. However, expression of these subunits has not been explored in the rat spinal cord. Here, we demonstrate presence of a dense plexus of GIRK1^+^ and -2^+^ neuronal processes and cell bodies in, mainly, lamina II in the rat dorsal horn, as well as in nerve terminals in the skin. The decrease in staining in the dorsal horn after axotomy and rhizotomy, suggests that GIRK^+^ processes not only originate from local neurons but also represent primary afferents, i.e. GIRK1 and -2 have a presynaptic localization. This is also supported by the co-localization of GIRK1 or -2 with VGLUT1 in a few boutons in the dorsal horn, since VGLUT1 in this region is only presented in, even if few, primary afferents [78]. Further support comes from the fact that the GIRKs accumulate around a ligation of the sciatic nerve, i.e. the channels are transported in axons.

A presynaptic localization has been shown in some studies [79, 80]. Ladera et al. reported, using immunogold electron microscopy, presence of GABA_B_ receptors and GIRK2 and -3 in presynaptic boutons in cerebral cortex, and a GABA_B_-mediated reduction in glutamate release that could be reversed by the GIRK channels blocker tertiapin-Q [80]. A similar situation exists in the cerebellum, here involving parallel fibers and, again, GABA_B_ receptors [11]. Taken together, GIRKs may play a role in presynaptic control of excitatory signaling also in the dorsal horn (and skin) and via such mechanisms influence pain signaling.

GIRK1-IR cell bodies were also found in other spinal layers, some multipolar neurons in lamina V and white matter and fusiform neurons in the deep layers. Thus, GIRK1^+^ neurons exhibit diverse morphological properties in deep dorsal horn layers. They may also represent projection neurons. In addition, an extensive expression of GIRK1-LI in the ventral horn was seen from lamina VI to X, some of which were co-localized with CGRP and thus represent motor neurons. Similarly, GIRK2-IR cells bodies were found in different layers of the spinal dorsal horn, but none in the ventral horn.

Inhibitory interneurons constitute 30–40% of neurons in rat lamina I–III, many of which serve an important anti-nociceptive function, and virtually all are GABAergic, some using glycine as co-transmitter [81–83]. Around half of the inhibitory interneurons in lamina I–II possess SST2A [52], all of which are GABA^+^ in lamina I–II of rat [84]. Here, we did not find any SST2A^+^ interneurons in rat lamina I–II that were GIRK1^+^, and only a few were GIRK2^+^. Thus, rat GIRK interneurons may in general, as in mouse, be excitatory. Also, GIRK channels may be of limited importance in pain signaling through somatostatin receptors at the spinal level, although GIRK3 and -4 should be considered. In agreement, GIRK channels influence signaling from myelinated low-threshold mechanical nociceptive afferents as well [85].

### Axonal transport of GIRK1 and -2

GIRK1- and -2-LIs were observed in control rat sciatic nerve at apparently comparable levels. This is somewhat surprising, since there are almost 10 times as many GIRK1^+^ as GIRK2^+^ NPs in the DRGs. Sciatic nerve ligation caused an accumulation of both GIRKs on the proximal and distal side of the lesion, indicating both antero- and retrograde axonal transport of the channels. We also found GIRK1^+^ and GIRK2^+^ fibers in the dermis layer of hind paw. Twenty-one days after dorsal rhizotomy, there was a reduction of both GIRK1- and -2-IR processes in the spinal dorsal horn, providing further support also for central centrifugal transport from the DRG cell bodies.

### Regulation of GIRK1 and -2 by peripheral nerve injury

Persistent hyperexcitability of peripheral nociceptors caused by changes in receptor and ion channel activity is an important mechanism in chronic pain conditions [33, 86–88]. In the peripheral sensory system, ion channels like Na^+^ and Ca^2+^ channels, contribute to excitation of sensory neurons under chronic pain conditions, either by up-regulation or enhancement of activity [89, 90]. K^+^ channels set the resting membrane potential in neurons, and thus control the excitability of sensory neurons under physiological conditions [1]. Here, we found that nerve injury caused a significant down-regulation of the K^+^ channels GIRK1 and -2 in DRG neurons, both at the mRNA and protein levels, as well as in the dorsal horn, 14 days after peripheral nerve injury.

These results are in line with previous studies showing that other types of K^+^ channels also are down-regulated after various types of peripheral nerve injury [31, 33]. An immunohistochemical analysis of rats subjected to the Chung model of neuropathic pain has demonstrated expression of various Kv1 family subunits in distinct DRG neuron populations, including nociceptors, as well as a distinct reduction in channel protein levels [55]. Moreover, axotomy decreased mRNA levels for certain Kv1 subunits with up to 80%, in parallel decreasing K^+^ currents [54]. These findings have in general been interpreted to show that a reduction of voltage gated K^+^ channel activity after trauma causes nerve hyperexcitability, and thus may contribute to peripheral and perhaps also central mechanisms underlying neuropathic pain [33]. The present and other studies suggest that a similar scenario then can be advanced for GIRK1 and -2. The conductance of inwardly rectifying potassium channels is greatest at membrane potentials close to the resting voltage [1]. Hence, GIRK channels are thought to be a key regulator of excitability, increasing the stimulation current needed for eliciting an action potential. In contrast, other K^+^ channels which open at more depolarized voltages, have less impact on the firing threshold [91].

It has been suggested that many of the changes in protein expression in DRGs after nerve injury serve to counteract pain [47], for example, the up-regulation of the inhibitory transmitters galanin and NPY, and the down-regulation of excitatory neuropeptides CGRP and substance P result in attenuated pain signaling in the dorsal horn. Thus, this apparently contrasts the consequences of the decreased expression of various K^+^ channels which enhance pain sensation. However, it may be speculated that a reduced number of presynaptic GIRK1 channels may allow facilitated release of up-regulated pain-inhibiting molecules like galanin from primary afferents in the dorsal horn and thus contribute to pain relief. In summary our data show a window of opportunities to restore or enhance inhibitory signaling by locally produced analgesic peptides by restoring or enhancing downstream GIRK mediated suppression of excitability.

## Concluding remarks

We show that both GIRK1 and GIRK2 channels are expressed in DRG neurons, where GIRK2 appears to be selective for small unmyelinated non-peptidergic and large myelinated neurons, contrasting the extensive expression of GIRK1 (~70% of all NPs) in different types on neuron populations. Since GIRK1 needs a partner to become functional, and less than 10% of the NPs are GIRK2^+^, a combination with GIRK4 appears as an attractive alternative. However, GIRK3 also merits investigation. Nerve injury caused a significant down-regulation of both GIRK1 and -2 at mRNA and protein levels. Considering the importance of K^+^ channels in setting the resting membrane potential, this down-regulation could contribute to the hyperexcitability after nerve injury and therefore to neuropathic pain.

## Methods

### Animals

The experiments were performed on adult male Sprague–Dawley rats (200–250 g, B&K Universal, Stockholm, Sweden). All animals were kept under standard conditions on a 12-h day/night cycle with free access to food and water. The studies were approved by the local Ethical Committee for animal experiments (Norra Stockholms djursförsöksetiska nämnd). Efforts were made to minimize the number and discomfort of the animals throughout the study.

### Surgeries

Animals were deeply anesthetized with isoflurane. Unilateral, complete transection (axotomy) of the sciatic nerve was performed as previously described [92]. All of the operated animals were allowed to survive for 14 days after surgery. Unilateral dorsal rhizotomy and sciatic nerve ligation were carried out according to published procedures [93, 94]. Briefly, the skin and muscles were incised to expose the vertebral laminae under deep anesthesia. Then, the L2–5 dorsal roots were transected, and muscle and skin were sutured. All the animals were allowed to survive for 21 days after surgery. With regard to the nerve ligation, the animals were sacrificed after 10 h.

### Immunohistochemistry

All animals were deeply anesthetized with sodium pentobarbital (50 mg/kg, i.p.), then transcardially perfused with 50 ml warm saline (0.9%; 37°C), followed by 50 ml of a mixture of 4% paraformaldehyde and 0.4% picric acid in 0.16 M phosphate buffer (pH 7.2, 37°C), and then by 250 ml of the same, but ice-cold fixative. L4-5 DRGs and corresponding segments of spinal cord were dissected out and post-fixed in the same fixative for 90 min at 4°C. Specimens were stored at 4°C for 2 days in 10% sucrose in phosphate buffered saline (PBS, 0.1 M, pH 7.4) containing 0.01% sodium azide (Sigma, St. Louis, MO, USA) and 0.02% bacitracin (Sigma) as preservatives. Tissues were embedded in OCT compound (Tissue Tek, Sakura, Leiden, Netherland), sectioned in a cryostat (Microm, Heidelberg, Germany) at 12 μm (DRGs), 14 μm (sciatic nerve and skin of hind paw) or 20 μm (spinal cord) thickness and mounted onto slides (SuperFrost Plus, Thermo, Waltham, America). Immunoreactivities were visualized using the tyramide signal amplification system (TSA Plus; NEN Life Science Products, Boston, MA, USA) [46]. For double-staining, we first performed TSA Plus staining, followed by indirect immunohistochemistry [95].

The primary anti-GIRK1 and -GIRK2 antibodies were purchased from Alomone labs (Jerusalem, Israel). The polyclonal antibodies against GIRK1 (P63250: Mouse 437–501 aa, intracellular C terminal) and GIRK2 (P48542: Mouse 374–414 aa, intracellular C terminal) were both raised in rabbits. The sequence of the GIRK1 immunogen is identical between mouse and rat, and with regard to GIRK2 highly conserved between these two species (40/41 amino acid residues identical). The antibody information above is from Alomone labs. Specificities of these two antibodies have been documented in previous studies, also using knock-out mice [38, 96], and were here further confirmed by incubating sections of DRG or spinal cord with antiserum pre-adsorbed with the homologous antigen (Alomone labs). This resulted in no detectable fluorescent signal (Additional file 1: Figure S1).

Details of all primary antibodies used are provided in Table 1. Antibodies were diluted in PBS containing 0.2% (w/v) bovine serum albumin (Sigma), 0.03% TritonX-100 (Sigma) and 0.1% (w/v) sodium azide (Sigma). To detect IB4^+^ neurons, the sections were incubated with IB4 from *Griffonia simplicifolia* I (GSA I; IB4; 2.5 g/ml; Vector Laboratories, Burlingame, CA, USA) followed by incubation with a goat anti-GSA I antiserum (1:2,000; Vector Laboratories) [97].

**Table 1:** Primary antibody list

Antibody	Host	Experiment	Dilution	Supplier/catalogue #	Characterization
GIRK1	Rabbit	IHC(TSA)	0.17 μg/ml	Alomone Labs/APC-005	Nockemann et al. [22]
		IHC(Coons)	0.75 μg/ml		
		WB	1:400		Marker et al. [37]
		IHC(TSA)	0.2 μg/ml	Alomone Labs/APC-006	Nockemann et al. [22]
GIRK2	Rabbit	IHC(Coons)	0.8 μg/ml		
		WB	1:400		Marker et al. [37]
CGRP	Rabbit	IHC(Coons)	1:2,000	S.I. Grigis	Orazzo et al. [98]
NF200	Mouse	IHC(Coons)	1:400	Sigma/N0142	Perry et al. [99]
Galanin	Rabbit	IHC(TSA)	1:4,000	E. Theodorsson	Theodorsson and Rugarn [100]
nNOS	Sheep	IHC(Coons)	1:400	P. Emson	Herbison et al. [101]
NPYY1	Rabbit	IHC(Coons)	1:400	J. Walsh, H. Wong	Zhang et al. [102]
SST1	Rabbit	IHC(Coons)	1:1,000	S. Schulz	Imhof et al. [103]
SST2A	Rabbit	IHC(TSA)	1:100	S. Schulz	Imhof et al. [103]
VGLUT1	Rabbit	IHC(Coons)	1:400	R.H. Edwards	Landry et al. [104]
PGP9.5	Rabbit	IHC(Coons)	1:1,600	Ultra Clone	Wang et al. [105]
PV	Rabbit	IHC(Coons)	1:400	Swant/PV 25	Mulder et al. [95]
CR	Rabbit	IHC(Coons)	1:400	Swant/7699	Mulder et al. [95]
CB	Rabbit	IHC(Coons)	1:400	Swant/CB 38	Mulder et al. [95]
Scgn	Mouse	IHC(Coons)	1:1,000	Atlas/13B8	Mulder et al. [106]
β-actin	Mouse	WB	1:5,000	Sigma/A5441	Amici et al. [107]

### Western blot analysis

Fourteen days after surgery, ipsi- and contralateral L4–5 DRGs (n = 4) were removed, immediately put on dry ice, separately pooled and placed in lysis buffer containing protease inhibitor (P8340; Sigma), and then sonicated. Lysates were centrifuged at 12,000 rpm for 30 min at 4°C. The supernatant was collected for western blot analysis. Protein concentration was measured by Bradford’s Assay (Bio-Rad, Hercules, CA, USA). Laemmeli sample buffer containing around 20 g of protein was loaded in each lane, separated on 10% SDS-PAGE gel, and transferred to polyvinylidene fluoride (PVDF) membranes (Millipore, Hemel, Hempstead, UK). The membranes were blocked with 5% non-fat dry milk in TBS with 0.1% Tween-20 for 1 h at room temperature (RT) and incubated overnight at 4°C with an antibody against GIRK1 (1:400; APC-005) or GIRK2 (1:400; APC-006). The membranes were incubated with HRP-conjugated secondary antibodies for 1 h at RT (1:10,000; DAKO), and exposed to ECL solution (Bio Rad) for 5 min. The membranes were stripped and re-probed for β-Actin (mouse monoclonal, 1:5,000; Sigma) as loading control.

### In situ hybridization

ISH using oligoprobes was carried out as described previously [108]. Commercially available oligonucleotide probes (CyberGene, Stockholm, Sweden) were used: (1) GIRK1: TGGGGACTTCAAAGGTTGCATGGAACTGGGAGTAATCGA; (2) GIRK2: GTGCTTTTCCTTGTGGTGGACAGGGTAGGTTCACTTCATC complementary to nucleotide sequences of rGIRK1 [GenBank: NM_031610.3], rGIRK2 [GenBank: NM_013192.2] mRNAs, respectively [109, 110]. Briefly, probes were radioactively labelled with ^33^P-dATP and purified with G50 DNA chromatography column (GE Healthcare). Only probes with radioactivity above 1 × 10^5^ CPM/μl were used. Twelve μm-thick DRG sections were air-dried and incubated with a hybridization solution containing 2 ng of labelled probe/slide at 42°C overnight. After hybridization, sections were washed in 1 × SSC 20 min/time at 55°C, 4 times. Then, sections were dehydrated as follows: in distilled water for 10 s, 60% ethanol for 15 s, and 95% ethanol for 15 s. The ^33^P-dATP-labelled sections were exposed for 6 weeks after dipping in NTB emulsion solution (Carestream Healthcare Inc, NY, USA).

### Image analysis and quantification

The stained slides were captured using a 10× (Plan-APOCHROMAT 10×/0.45) or 20× (Plan-APOCHROMAT 20×/0.8) primary objective on a VSlide slide-scanning microscope (Metasystems, Altlußheim, Germany) equipped with filter sets for DAPI (EX350/50–EM470/40), FITC (EX493/16–EM527/30) and Cy5 (EX630/20–647/long pass). Individual field-of-view images were stitched to produce images of entire DRG and spinal cord sections with microscopic resolution. Images were analyzed with MetaViewer software (Metasystems) separately and merged to evaluate possible co-existence through channel operations. Photographs were taken with a Nikon Coolpix 5000 digital camera (Nikon, Tokyo, Japan).

To determine the percentage of GIRK1^+^ and GIRK2^+^ NPs in normal and contra-/ipsilateral DRGs, every 4th or 6th 12 μm-thick section was selected for counting. All countings were conducted with MetaViewer software (Metasystems). Total number of DRG NPs was counted using Hoechst staining. The number of GIRK1^+^ or GIRK2^+^ NPs was divided with total NPs, and percentages were calculated. Four sections from each DRG in four animals per group were included in the analysis. To determine the percentages of co-existence of GIRK1 and -2 with neuronal phenotypic markers including CaBPs, DRG sections from five to ten animals were selected and counted as previously described [111]. The cross-sectional area and intensity (mean gray value) of GIRKs^+^ neurons were collected from Image J software (National Institutes of Health). Only GIRKs^+^ neurons with a clear nucleus were selected.

Image J software was also used to measure the intensities of GIRK1- and GIRK2-LI in contra- and ipsilateral spinal cord lamina II after nerve injury. Briefly, images extracted from MetaViewer (Metasystems) were opened with Image J, the area of lamina II was drawn using outline tool, then the mean pixels were calculated by “Measure” function. Background intensity was subtracted in each spinal cord.

### Statistical analyses

All data were expressed as Mean ± SEM. Differences between the percentage of GIRK1- and -2-IR NPs in contra- and ipsilateral DRGs, and intensities of GIRK1- and -2-LIs in spinal contra- and ipsilateral lamina II were evaluated by unpaired Student’s *t* test. P < 0.05 was taken as the criterion for statistical significance.

## Additional file

Additional file 1:
**Figure S1.** Absorption test for anti-GIRK1 and -2 antibodies in DRGs and spinal cord. Immunohistochemical images show GIRK1 and -2 staining in control DRGs (**A**, **E**) and spinal cord (**C**, **G**). After pre-absorption of GIRK1 and GIRK2 antiserum with the corresponding antigen peptide, no signal can be detected in DRGs (**B**, **F**) or spinal cord (**D**, **H**). Scale bars indicate 40 μm (**A**, **B**, **E**,** F**), 500 μm (**C**, **D**, **G**, **H**).
