# Supplementary figures and images for: G protein-gated inwardly rectifying potassium channel subunits 1 and 2 are down-regulated in rat dorsal root ganglion neurons and spinal cord after peripheral axotomy
Source: Mol Pain. 2015 Jul 22;11:44. doi: 10.1186/s12990-015-0044-z (PMC4511542; doi:10.1186/s12990-015-0044-z)

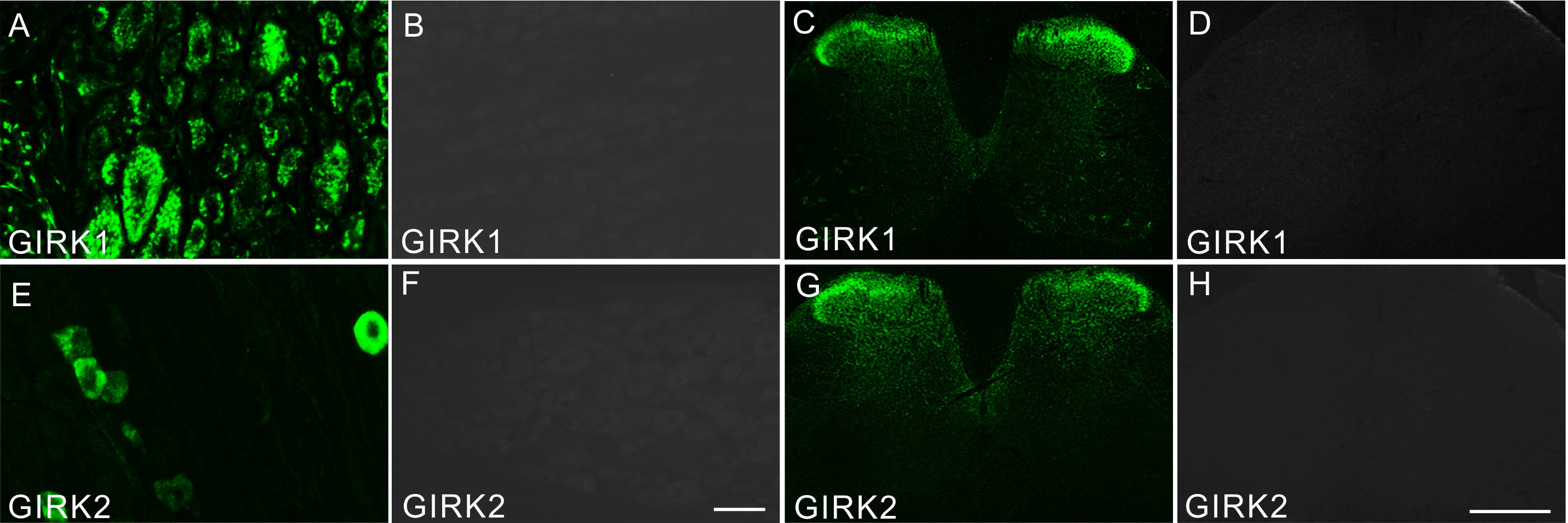

Supplement: Additional file 1: — Figure S1. Absorption test for anti-GIRK1 and -2 antibodies in DRGs and spinal cord. Immunohistochemical images show GIRK1 and -2 staining in control DRGs (A, E) and spinal cord (C, G). After pre-absorption of GIRK1 and GIRK2 antiserum with the corresponding antigen peptide, no signal can be detected in DRGs (B, F) or spinal cord (D, H). Scale bars indicate 40 μm (A, B, E, F), 500 μm (C, D, G, H). [file 12990_2015_44_MOESM1_ESM.tif]
